# The Moderating Effects of Disability on Mobile Internet Use Among Older Adults: Population-Based Cross-sectional Study

**DOI:** 10.2196/37127

**Published:** 2022-04-04

**Authors:** Eunjin Yang, Kyung Hee Lee

**Affiliations:** 1 Mo-Im Kim Nursing Research Institute Yonsei University College of Nursing Seoul Republic of Korea

**Keywords:** older adults, people with disabilities, digital divide, mobile phone use

## Abstract

**Background:**

The preferred devices to access the internet are changing from personal computers to mobile devices, and the number of older adults with or without disabilities is rapidly increasing in an aging society. However, little is known about the moderating effects of disability on mobile internet use among older adults.

**Objective:**

This study aimed to examine the levels of mobile internet use and factors associated with this use among older adults according to their disabilities. In addition, moderating effects of disability on mobile internet use were investigated.

**Methods:**

This study consisted of a secondary data analysis using the 2020 Digital Divide Survey conducted in South Korea. The single inclusion criterion was participants being aged 55 years or older; accordingly, 2243 people without disabilities and 1386 people with disabilities were included in the study. Multiple regression analyses considering complex sample designs were conducted to identify mobile internet use factors and to test the moderating effects of disability on mobile internet use.

**Results:**

Older adults with disabilities used mobile internet less than older adults without disabilities. However, disability status had moderating effects on the relationships between mobile internet use and (1) operational skills regarding mobile devices (B=0.31, *P*=.004), (2) internet use skills (B=1.46, *P*<.001), (3) motivation to use digital devices (B=0.46, *P*=.01), and (4) attitude toward new technology (B=0.50, *P*=.002). The results revealed that these positive relationships were stronger among older adults with disabilities than among adults without disabilities.

**Conclusions:**

Although older adults and people with disabilities are considered vulnerable populations regarding technology adoption, disability creates a stronger association between several determinants and actual mobile internet use. Therefore, policy makers and practitioners should pay attention to older adults with disabilities to deliver appropriate information-literacy education. Older adults with disabilities could be the primary beneficiaries of mobile services and new technology.

## Introduction

During the COVID-19 pandemic, most offline activities and services were transferred online according to the regulations and guidelines for infectious disease response [1,2]. In particular, South Korea implemented principles and policies based on information and communications technology (ICT) to respond to infectious diseases [3]; because of this, digital transformation is accelerating in the country. Use of ICT is becoming more crucial in this highly connected information society. In addition to these situations, the digital divide—defined as the gap between ICT users and nonusers in terms of access to or use of ICT based on their economic, regional, physical, and social factors—is becoming serious [4]. Discourse around the digital divide in society has changed the issue from material access to that of effective use of technology [5,6]. South Korea has already achieved a highly connected network society through high internet penetration; therefore, digital divide issues, such as skills and benefits from information use, are emerging beyond material access issues [7].

Among various populations, older adults are less likely to adopt, diffuse, or use ICT [8]. Therefore, with the prevalence of non–face-to-face activities and social services, older adults who do not use the internet are more likely to be disadvantaged [9]. Older adults who are nonusers of the internet face double the burden, that of digital and social exclusion. They can become easily isolated, feel lonely, and face many difficulties in using the digital technologies that pervade daily life compared to internet users [10]. In addition, older adults generally have poor health status and are prone to disabilities, such as brain lesions, visual impairment, and hearing impairment. These disabilities may impact their technology adoption and mobile internet usage [11].

In terms of populations with disabilities, there are conflicting opinions regarding technology use. Previous studies reported that people who have disabilities experience the digital divide more frequently [12]. On the other hand, there exists a differing view that people with disabilities want to use online information and digital technology to make their voices heard and participate in the online community [13]. Once people with disabilities can connect to the internet and mobile accessibility issues are resolved, the online world can potentially become a physical barrier–free environment for them [14]. Therefore, digital technology, including mobile internet use, is considered both a challenge and an opportunity for people with disabilities [15].

Mobile internet use among older adults and people with disabilities has risen over time, and these trends increased dramatically during the COVID-19 pandemic [16,17]. In addition, the main devices used to access and use the internet are changing from personal computers to smart mobile devices. Approximately 91.5% of the total population aged 3 years and above use mobile internet, and 93.1% of those aged 6 years and above possess smartphones in South Korea [18]. This means that everyone who owns a smart mobile device can use ICT services, regardless of the time and place. Mobile internet use significantly affects people’s chances to obtain information and build social capital. Therefore, there is a gap in this regard between mobile internet users and nonusers [19].

Previous studies revealed many factors associated with mobile internet use in various dimensions, including social, cultural, personal, material, and motivational aspects [20]. According to van Dijk’s digital divide theory, motivation, materials, and skills are continuously and recursively associated with digital information usage and participation in society [6]. Moreover, skills, attitudes, motivation, and internet efficacy are closely linked to the use of internet information [20]. Many studies have investigated the relationship between these associated factors and internet use among people with disabilities and older adults [16,20]. Some studies have reported that people with disabilities are less likely to use the internet than those without disabilities [21,22], and they have compared the digital divide among people with disabilities and those without [15,21]. One study reported a moderating effect of disability on smartphone online activities; however, it examined all age groups, and the results showed that the moderating effects of disability existed in the relationships between smartphone online activities and the attitude and social support factors [16]. However, older adults may show different patterns of mobile internet usage compared to other age groups, and the relationship between mobile internet use and its associated factors could differ according to their health and disability status [23]. To fill in this research gap, research regarding older adults’ mobile internet use according to their disabilities is needed. Even if older adults were less likely to use the internet than younger adults, many of their health care needs, such as reliable health information, could be addressed by information disseminated by the internet. Therefore, before preparing strategies to facilitate older adults’ mobile use, it may be helpful to understand the relationships between several factors associated with technology and the adults’ actual mobile internet use. Accordingly, this study examined the levels of mobile internet use and its related factors among older adults based on their disabilities. In addition, it investigated the moderating effects of disability on mobile internet use.

## Methods

### Data and Study Participants

We used data from the 2020 Digital Divide Survey, which was conducted in South Korea from September to December 2020 through face-to-face interviews using structured questions. This survey was managed by the Ministry of Science and ICT and the National Information Society Agency; it has been implemented annually since 2002. The survey examined the effectiveness of policy to bridge the digital divide between the general population and vulnerable populations, such as people with disabilities, older adults, and North Korean defectors, among others. These data were collected using a complex survey design. Older adults were recruited through stratified proportional sampling; people with disabilities were recruited through proportional sampling by group characteristics (eg, age, gender, type of disability, and geographic area). In this study, people with disabilities were defined as those who were registered as such according to the national registration process after medical diagnosis. In terms of disability types, physical, brain lesion, visual, hearing, and speech disabilities were included in this survey among the 15 types of disabilities defined in the Enforcement Decree of the Act on Welfare of Persons with Disabilities of South Korea [24]. The participants’ only inclusion criterion in this study was being aged 55 years or above; therefore, of the 2200 people with disabilities, 871 (39.59%) who were aged below 55 years were excluded, leaving 1329 people. Although the survey for older adults did not specifically target people with disabilities, a question assessing the presence of disability was included in the questionnaire. However, details regarding the types of disability were not assessed. Therefore, among 2300 older adults without disabilities, 57 people who answered as having a registered disability, based on legal standards, were reclassified as people with disabilities. In the end, 2243 people without disabilities and 1386 people with disabilities were included in this study.

### Variables

#### Dependent Variable

The level of mobile internet use was calculated based on three points:

1. Whether people used the internet recently in the past month or not and days of use (1 item).
2. Whether people used a variety of internet services (13 items, including general search, email, online content services, social networks and cloud services, and information searches regarding daily life).
3. Whether people used advanced internet services (12 items, including information generation and sharing, online networking, and participation in social activities and the economy).

Items for points 2 and 3 were measured on a 4-point scale. The level of mobile internet use was calculated by considering item weights (ie, 0.4, 0.4, and 0.2, respectively, for each of the three points). The score was calculated based on the guidance provided by the National Information Society Agency, and the total score ranged from 0 to 100.

#### Independent Variables

Independent variables included operational skills regarding mobile devices, internet use skills, motivation to use digital devices, and attitude toward new technology. Operational skills regarding mobile devices were defined as skills for operating mobile device hardware and software [25,26]; these were investigated using seven items on a 4-point scale, ranging from 1 (strongly disagree) to 4 (strongly agree). The seven items were as follows: (1) configuring mobile devices; (2) accessing Wi-Fi networks; (3) transferring files from mobile devices to personal computers; (4) transferring files or photos from the participants’ mobile devices to those of other people; (5) installing, deleting, and updating mobile apps; (6) dealing with malware in mobile devices; and (7) creating documents on mobile devices. The possible score ranged from 7 to 28, and Cronbach α was .94 in this study.

Skills for internet use, also called strategic internet skills, are comprised of the capacity to access and manage information using digital devices to reach particular goals [25,26]. They were measured using four items on a 4-point scale, ranging from 1 (strongly disagree) to 4 (strongly agree). The items were as follows:

1. I can connect and communicate with others through the internet and cooperate with them for problem solving and work.
2. I can exchange opinions about political and social issues and problems through the internet and participate in various activities, such as discussion, donation, and solving of public problems.
3. I can protect myself and others from risk factors associated with internet use, such as leaking of private information.
4. I can understand others’ opinions, accept different views, and use the internet responsibly, while not accessing illegal media or infringing on other people’s rights.

The total score ranged from 4 to 16, and the Cronbach α was .88 in this study.

The scale for motivation to use digital devices included five items on a 4-point scale, ranging from 1 (strongly disagree) to 4 (strongly agree). Items were related to participants’ (1) eagerness to obtain information using digital devices, (2) wish to become acquainted with many persons through digital devices, (3) wish to be entertained through digital devices, (4) need for self-development, and (5) need to express one’s opinion through digital devices. The total score ranged from 5 to 20, and the Cronbach α was .88 in this study.

The scale for attitude toward new technology comprised six items on a 4-point scale, ranging from 1 (strongly disagree) to 4 (strongly agree). The items were as follows:

1. I tend to adapt well to new technology and products.
2. I am confident in using new technology and products by myself.
3. When I use new technology and products, I do well as compared with others.
4. I think digital technology is essential to continue economic activities.
5. I try to learn new technology actively.
6. I think I am a lifetime learner and enjoy the classes needed to learn new technology.

The total score ranged from 6 to 24, and the Cronbach α was .88 in this study.

#### Covariates

The demographic characteristics of age (continuous), gender (female vs male), education (below vs above high school), living arrangements (living alone vs with others), household income (below vs above ₩4,000,000/month; a currency exchange rate of ₩1=US $0.00082 is applicable), and living areas (urban vs rural) were selected as covariates [12,20].

### Statistical Analysis

Data analysis was carried out using SPSS Statistics for Windows (version 26.0; IBM Corp). Data were analyzed using complex sampling analysis considering sample weight. Comparison of demographic characteristics and variables between older adults with disabilities and those without disabilities was conducted using the *t* test (2-tailed) and Rao‐Scott chi‐square tests. A multiple regression analysis was applied to examine the association between independent variables and mobile internet use. To explore the moderating effect of disability, first, the covariates, independent variables (ie, operational skills, internet use skills, motivation, and attitude), and moderator (ie, disability) were entered into the model as a block. Then, four interaction terms (ie, operational skills × disability, internet use skills × disability, motivation × disability, and attitude × disability) were entered into each model; therefore, four separate models were created. Statistical significance was set at the .05 level in all analyses. The slope of the moderation effect of disability was presented using the EasyFlow Statistics macro in Excel [27].

### Ethical Considerations

The Korean Statistics Act guaranteed information protection for all participants, and participants were notified about this before the survey. This study was a secondary analysis study using publicly available data; therefore, the Institutional Review Board (IRB) approved this study as an exempt study (IRB No. 4-2021-1743). All study processes were performed according to the relevant guidelines and regulations.

## Results

### Characteristics and Variables of Groups With Disabilities and Those Without Disabilities

Participants’ general characteristics and independent variables based on disability status are summarized in Table 1. In terms of disability types among the group of persons with disabilities, over half of them (61.85%) were physical disabilities. Most demographic characteristics differed between the two groups. The mean age of older adults without disabilities (mean 66.54, SD 0.18 years) was higher than that of older adults with disabilities (mean 62.60, SD 0.14 years), and there were more males in the group of older adults with disabilities. In terms of education, a higher proportion of older adults without disabilities had a high educational level (81.43%; ie, high school education and above) compared with those with disabilities (50.54%). As for living arrangements, 16.86% of those with disabilities lived alone, while 8.58% of those without disabilities lived alone.

Regarding independent variables, operational skills regarding mobile devices (*P*<.001), internet use skills (*P*<.001), motivation to use digital devices (*P*=.04), and attitude toward new technology (*P*<.001) were higher among older adults without disabilities compared to those with disabilities. Mobile internet use was also higher in the group without disabilities than in the group with disabilities (*P*<.001).

**Table 1:** Demographic characteristics and variables of the participants (N=3629).

Characteristic or variable		Participants with disabilities (n=1386)	Participants without disabilities (n=2243)	*t* test^a^ (*df*=4498)	*F* test^a,b^ (*df*=1, 3628)	*P* value^a^
**Type of disability, n (%)^c^**						
	Physical disability	862 (61.85)	N/A^d^	N/A	N/A	N/A
	Brain lesion	151 (10.78)	N/A	N/A	N/A	N/A
	Visual impairment	153 (11.36)	N/A	N/A	N/A	N/A
	Hearing impairment	133 (9.49)	N/A	N/A	N/A	N/A
	Speech disability	30 (2.35)	N/A	N/A	N/A	N/A
	Not reported	57 (4.17)	N/A	N/A	N/A	N/A
Age (years), mean (SD)		62.60 (0.14)	66.54 (0.18)	17.196	N/A	<.001
**Gender, n (%)^c^**						
	Female	494 (35.27)	1199 (53.43)	N/A	95.07	<.001
	Male	892 (64.73)	1044 (46.57)			
**Educational level, n (%)^c^**						
	Below high school	724 (49.46)	414 (18.57)	N/A	333.32	<.001
	High school and above	662 (50.54)	1829 (81.43)			
**Living arrangements, n (%)^c^**						
	Living alone	231 (16.86)	192 (8.58)	N/A	50.34	<.001
	Living with others	1155 (83.14)	2051 (91.42)			
**Household income (₩^e^/month), n (%)^c^**						
	<4,000,000	1269 (90.84)	1263 (56.35)	N/A	326.85	<.001
	≥4,000,000	117 (9.16)	980 (43.65)			
**Living area, n (%)^c^**						
	Urban	1255 (92.27)	2083 (92.85)	N/A	0.45	.50
	Rural	131 (7.73)	160 (7.15)			
Operational skills regarding mobile devices^f^, mean (SE)		14.37 (0.18)	15.56 (0.12)	5.44	N/A	<.001
Internet use skills^g^, mean (SE)		7.02 (0.08)	7.76 (0.06)	7.01	N/A	<.001
Motivation to use digital devices^h^, mean (SE)		12.05 (0.11)	12.33 (0.07)	2.05	N/A	.04
Attitude toward new technology^i^, mean (SE)		12.73 (0.11)	13.56 (0.09)	5.78	N/A	<.001
Mobile internet use^j^, mean (SE)		40.05 (0.93)	44.08 (0.61)	3.64	N/A	<.001

^a^The *t* test (2-tailed), *F* test, and *P* values for a group are reported in the top row of that group.

^b^*P* values related to values in this column were determined using the Rao-Scott chi-square test.

^c^Percentages are weighted.

^d^N/A: not applicable; this measure did not apply to this group.

^e^A currency exchange rate of ₩1=US $0.00082 is applicable.

^f^Scores for operational skills ranged from 7 to 28.

^g^Scores for internet use skills ranged from 4 to 16.

^h^Motivation scores ranged from 5 to 20.

^i^Attitude scores ranged from 6 to 24.

^j^Mobile internet use scores ranged from 0 to 100.

### Factors Associated With Mobile Internet Use

Multiple regression analysis was performed to examine the factors associated with mobile internet use among older adults. As Table 2 shows, age, household income, living areas, operational skills regarding mobile devices, internet use skills, motivation to use digital devices, and attitude toward new technology were positively related to mobile internet use. Disability status was not significantly associated with mobile internet use in model 1. The *R*^2^ value of model 1 is 0.705.

**Table 2:** Factors associated with mobile internet use (N=3629).

Factor	Model 1 values		
	B (SE)	*t* test^a^ (*df*=4498)	*P* value
Constant	12.28 (4.16)	2.95	.003
Age	–0.58 (0.05)	–12.46	<.001
Gender (reference: female)	–0.83 (0.61)	–1.36	.17
Educational level (reference: below high school)	0.83 (0.81)	1.03	.30
Living arrangements (reference: living alone)	0.68 (0.91)	0.75	.46
Household income (reference: <₩4,000,000^b^/month)	–1.38 (0.68)	–2.04	.04
Living area (reference: rural)	–1.77 (0.90)	–1.97	.049
Disability (reference: no disability)	–1.31 (0.78)	–1.67	.10
Operational skills regarding mobile devices	2.49 (0.10)	25.60	<.001
Internet use skills	1.85 (0.17)	10.91	<.001
Motivation to use digital devices	0.89 (0.13)	6.62	<.001
Attitude toward new technology	0.56 (0.12)	4.88	<.001

^a^The *t* test was 2-tailed.

^b^A currency exchange rate of ₩1=US $0.00082 is applicable.

### Moderating Effects of Disability on Mobile Internet Use

The regression model to examine the moderating effect of disability indicated that disability status enhanced the relationship between independent variables and mobile internet use. The positive relationship between operational skills regarding mobile devices and mobile internet use was stronger among older adults with disabilities than among those without disabilities (B=0.31, *P*=.004). These moderating effects were also found in the relationship between mobile internet use and internet use skills (B=1.46, *P*<.001), motivation to use digital devices (B=0.46, *P*=.01), and attitude toward new technology (B=0.50, *P*=.002; Table 3).

**Figure 1:**
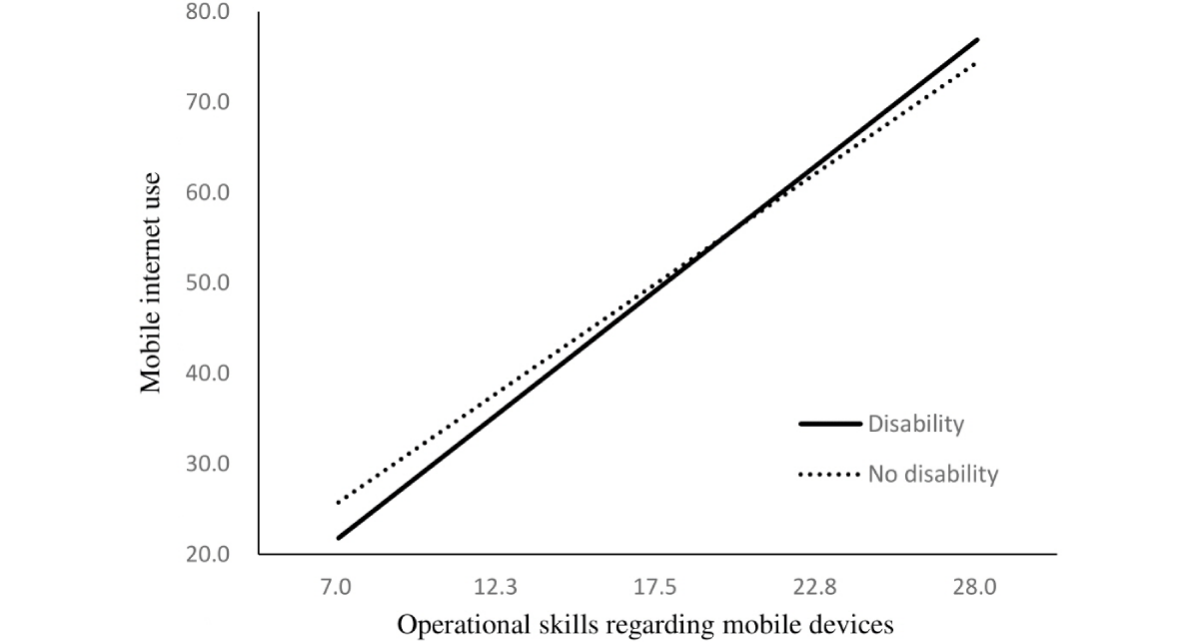
Simple slopes for the relationship between operational skills and mobile internet use. Respective scores are listed on the axes.

**Figure 2:**
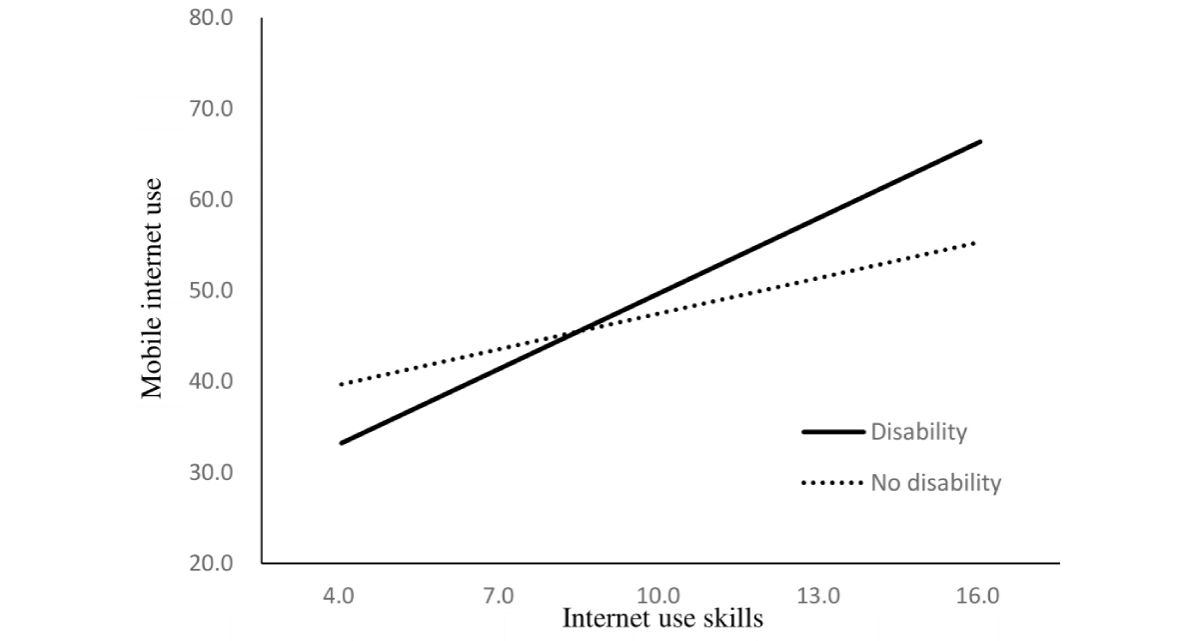
Simple slopes for the relationship between internet use skills and mobile internet use. Respective scores are listed on the axes.

**Figure 3:**
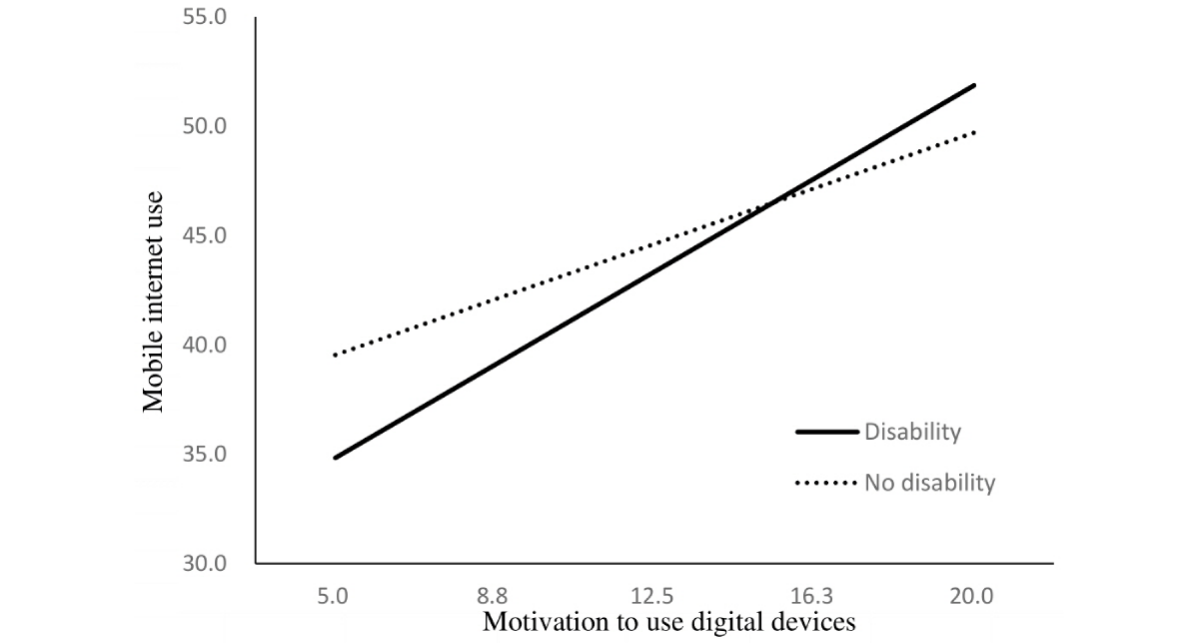
Simple slopes for the relationship between motivation and mobile internet use. Respective scores are listed on the axes.

**Figure 4:**
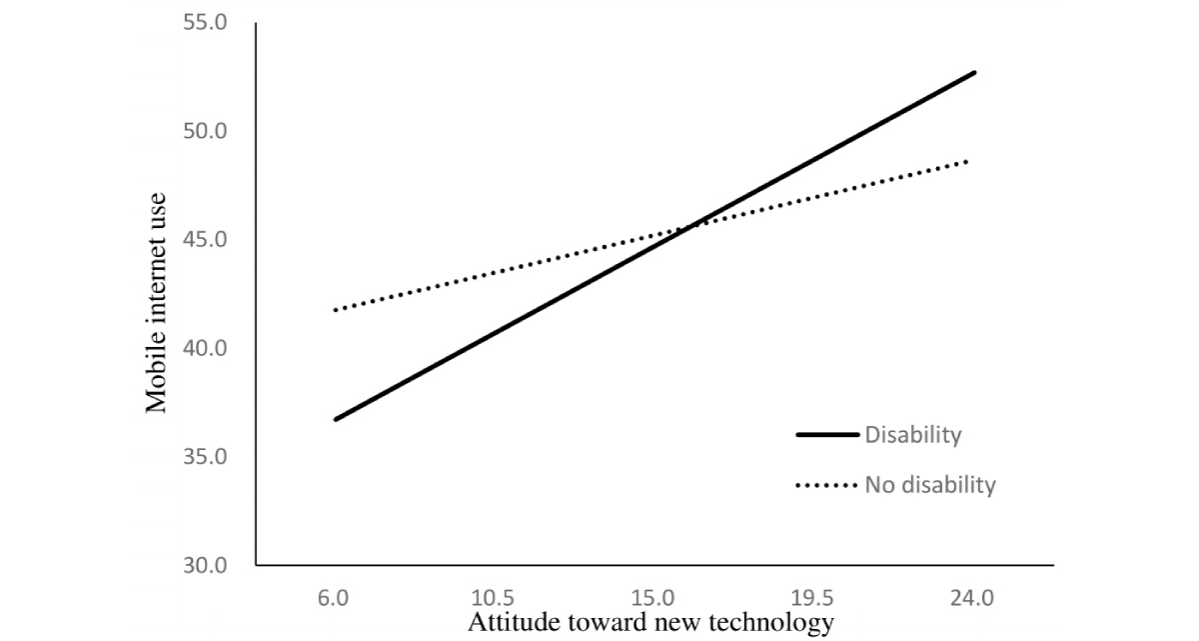
Simple slopes for the relationship between attitude and mobile internet use. Respective scores are listed on the axes.

The simple slopes of these relationships are presented in Figures 1 to 4.

**Table 3:** Moderating effects of disability (N=3629).

Model Number^a^	Model details	B (SE)	*t* test^b^ (*df*=4498)	*P* value	*R*^2^ (△*R*^2^)
2	Operational skills regarding mobile devices × disability	0.31 (0.11)	2.89	.004	0.706 (0.001)
3	Internet use skills × disability	1.46 (0.22)	6.64	<.001	0.710 (0.005)
4	Motivation to use digital devices × disability	0.46 (0.18)	2.53	.01	0.706 (0.001)
5	Attitude toward new technology × disability	0.50 (0.17)	3.03	.002	0.706 (0.001)

^a^Models 2 to 5 include all variables of model 1 from Table 2 (ie, age, gender, educational level, living arrangements, household income, living areas, disability, operational skills regarding mobile devices, internet use skills, motivation to use digital devices, and attitude toward new technology).

^b^The *t* test was 2-tailed.

## Discussion

### Principal Findings

This study examined the factors related to mobile internet use among older adults through a national representative sample in South Korea. The moderating effects of older adults’ disability status on their mobile internet use was also investigated. The bivariate analysis showed gaps in both sociodemographic characteristics and level of mobile internet use among older adults with disabilities and those without disabilities. The level of internet use among older adults with disabilities was lower than that among their counterparts. In addition, operational skills regarding mobile devices, internet use skills, motivation to use digital devices, and attitude toward new technology were also low among the group with disabilities.

Previous research has frequently reported differences in the level of internet use between people with disabilities and the general population [15,16]. Our study confirmed these gaps by focusing on mobile devices and the older adult population. The reason for these results may be that sociodemographic factors contributing toward access to and use of the internet [28], such as education, income, and living arrangements, among older adults with disabilities were poorer than those among older adults without disabilities. In addition, most mobile service and app designs and functions are less intuitive for older adults with disabilities, causing them to use the internet less than other populations [29,30]. The South Korean government releases web accessibility evaluation reports annually to ensure that technologically vulnerable populations, such as older adults and people with disabilities, can use the internet comfortably [31]. Although the government has developed standards for web accessibility and tries to bridge the digital divide, the standards and efforts to improve mobile web accessibility need to be reinforced beyond personal computer web accessibility.

In terms of factors related to mobile internet use, the sociodemographic characteristics of age, household income, and living area were statistically significant in this study, and our results were consistent with previous reports [20], except for disability. Unlike previous studies, which reported that disability is a determinant of internet use [12,15,21,28], this study found that disability is not statistically significant when it comes to using mobile internet. However, another study that focused on smartphones, and not personal computers, reported similar results as this study; it found that disability was not a significant factor related to mobile internet use when using the multiple regression model [16], which may be because of the types of devices targeted in each study. These results should be interpreted cautiously, as mobile devices are more appropriate than other devices for internet use by older adults, regardless of their disability status.

Therefore, mobile devices could provide many opportunities to older adults. Mobile device ownership, including smartphones, substantially increased among groups of people with disabilities and those without disabilities, and they became core tools for both groups. However, many information-literacy education courses for older adults provided at senior centers in South Korea focused on traditional personal computers for internet use [32]. From 2020 onward, the Korean government created digital competency centers as part of the digital inclusion policy, aiming for a rapid digital transformation. These are positive changes that can meet the need for mobile-based information-literacy education reflecting the trend of mobile device proliferation. Although these centers provide many courses [33], tailored interventions for older adults with disabilities and those without disabilities are still not enough and need to be expanded.

Other factors, such as operational skills, internet use skills, motivation to use digital devices, and attitude toward new technology, were also statistically significant in this study. All of these factors were reported as determinants of internet use [20], and our study confirmed that these relationships exist similarly among older adults with disabilities and those without disabilities. Many studies regarding the digital divide have verified that the predictors of internet use among people with disabilities are similar as those for people without disabilities [12,16]. This means that an integrated approach to increase mobile internet use and technology adoption among older adults, regardless of their disability status, is needed; this approach should consider common factors, such as skills, motivation, and attitude toward technology. Many countries, including Australia, Israel, New Zealand, Singapore, and the United Kingdom, have presented visions such as a digital readiness blueprint, digital initiative, and digital inclusion blueprint [34]. New Zealand’s digital inclusion blueprint identifies motivation, access, skills, and trust as important factors and raises a concern that people who do not possess these factors can be excluded from the digital world [35]. Therefore, these common crucial factors should be considered by policy makers and practitioners to ensure that older adults with or without disabilities become more engaged in mobile internet use and mobile technology.

It was interesting to find that although disability status was not statistically significant in the multiple regression model, all interaction effects were statistically significant when we entered the interaction between variables and disability in each model. Although older adults with disabilities use mobile internet less as compared with those without disabilities, if skills, motivation, and attitude are enhanced through appropriate technology education, their actual use of mobile internet could be facilitated. Today, mobile health care via mobile devices has become prevalent; however, these services have not usually been not targeted toward people with disabilities [36]. Our results thus shed light on the possibility that older adults with various health care needs could be valuable consumers and active users through an increase in their skills, attitudes, and motivation. These are positive signals regarding the disability digital divide; however, mobile accessibility issues and technical barriers should be resolved first [13,37]. Even though older adults have high levels of skills, motivation, or positive attitudes toward technology, if the universal design is not optimized for senior classes, older adults will not be able to use the technology appropriately. Older adults, especially those with disabilities, have many health information needs, and mobile internet use for health purposes could be helpful in not only managing their health but also improving their well-being and health-related outcomes [38]. Further, their mobile internet use can be advantageous, such as by increasing life satisfaction and ensuring social inclusion [39]. Therefore, apart from the common concerns regarding the digital divide of people with disabilities, policy makers and practitioners should pay attention to delivering mobile services and information education to older adults with disabilities, because older adults with disabilities could be the main beneficiaries of mobile services and new technology.

### Strengths and Limitations

A strength of this study is that it used nationally representative data from the 2020 Digital Divide Survey in South Korea, a country that has been ranked high in advanced ICT infrastructure among the Organisation for Economic Co-operation and Development countries. Further, this survey was conducted and managed by reliable professional survey firms and government institutions. Therefore, our findings are reliable and can be generalized to other older adults in South Korea. In addition, the findings will be helpful to other countries that have similar ICT infrastructure or technology environments.

The study also has several limitations. First, as the survey was conducted for only five types of disability, it does not encompass other types, such as developmental disabilities, internal organ impairments, and mental illnesses. Since internet use levels, barriers to internet use, and patterns of use could differ according to the various types of disability and severity [40], further studies that include all types of disability are needed. In addition, variables associated with mobile internet use were limited because this study involved secondary analysis.

### Conclusions

This study examined the levels of mobile internet use, factors associated with mobile internet use, and moderating effects of disability on mobile internet use among older adults. The findings have implications for policy makers, social workers, and health care providers who intend to deliver services using mobile internet and technology to improve older adults’ quality of life. Although older adults and people with disabilities are considered a vulnerable population, disability creates a stronger association between several determinants and actual mobile internet use. Therefore, appropriate information-literacy education to ensure that older adults engage in mobile internet use and mobile technology is needed. Accordingly, older adults with disabilities could be the main beneficiaries of mobile services and new technology.

## Abbreviations

ICT: information and communications technology
IRB: Institutional Review Board
NRF: National Research Foundation of Korea
